# Preserving the Open Access Benefits Pioneered by the Journal of Medical Internet Research and Discouraging Fraudulent Journals

**DOI:** 10.2196/16532

**Published:** 2019-12-23

**Authors:** Jeremy C Wyatt

**Affiliations:** 1 Department of Public Health and Primary Care University of Southampton Southampton United Kingdom

**Keywords:** open access, predatory journals, knowledge management, scientific journals, mobilizing computable knowledge, fraudulent journals

## Abstract

The *Journal of Medical Internet Research* (JMIR) was an early pioneer of open access online publishing, and two decades later, some readers and authors may have forgotten the challenges of previous scientific publishing models. This commentary summarizes the many advantages of open access publishing for each of the main stakeholders in scientific publishing and reminds us that, like every innovation, there are disadvantages that we need to guard against, such as the problem of fraudulent journals. This paper then reviews the potential impact of some current initiatives, such as Plan S and JMIRx, concluding with some suggestions to help new open-access publishers ensure that the advantages of open access publishing outweigh the challenges.

## Introduction

### Background

Scientific journals have a 355-year history, with the first, Le Journal des Sçavans, appearing in 1665, followed the same year by Philosophical Transactions of the Royal Society. Now there are more than 5000 scientific publishing companies with 25,000 journals publishing 1.5 million articles per year, generating revenues of US $25 billion [1]. Even more surprising are the changes in the last 20 years. Many *Journal of Medical Internet Research* (JMIR) authors and readers will have forgotten that, just two decades ago, JMIR was one of the very first scientific journals in any field to demonstrate the value and sustainability of open access publishing. JMIR remains the top-cited journal in the discipline of medical informatics, partly due to that bold decision by its editor, Gunther Eysenbach. Readers may have also forgotten the dark days of universal pay-for-access and the paper publishing model that preceded online publishing. Back then, a researcher periodically visited libraries to photocopy articles out of bound journals or waited weeks for an article from an obscure journal to arrive from an interlibrary loan service. So, it is timely to remind ourselves of the many advantages of open access publishing, consider some of the pitfalls, and explore some potentially fundamental changes in the publishing landscape over the next few years.

### Advantages of the Open Access Publishing Model in Medicine

Open access publishing brings many advantages for authors, researchers, research funders, publishers, the environment, and the patients (Textbox 1). For the author, publication in an open-access journal means that their work is 90% more likely to be read and 42% more likely to be downloaded as a PDF in the six months following publication than a comparable subscription-access article in the same journal [2]. The precise impact of open access publishing on citation rates is still debated [3]. For example, in a recent cohort study of 5835 medical journals, open access journals had a significantly higher CiteScore, percent cited, and source normalized impact per paper, but the non–open access journals had a higher scholarly output [4]. The broader societal impact of research is also significant, with higher Altmetric scores for open access articles [5]. As always, in cohort studies, association is hard to distinguish here from causation; however, the only trial which could answer this question rigorously by randomizing incoming articles to open or subscription-only access journals was carried out a decade ago in 11 physiology journals [2]. Thus, it may have limited generalizability today. This trial showed no real citation benefit for open access articles compared to comparable subscription-access articles during the year following publication [2].

For researchers, open access dramatically reduces the hassle of obtaining access to the full text, which is vital as openly accessible abstracts do not always fairly represent article contents [6]. However, structured abstracts have now potentially reduced this mismatch. For those who carry out and fund systematic reviews, such as guideline development groups, access to the full text to extract details of the study methods and results is particularly important. For this group, there was no apparent difference in the methodological quality of either primary or secondary research articles published in open access or subscription-only journals [7], at least in cancer epidemiology. For publishers, open access guarantees a steady flow of readers to their site, allowing them to experiment over time with different methods of indexing or presenting information in their journal using A-B testing and similar methods. This makes continuing quality improvement and enhancing reader impact much more straightforward than when articles sit behind a paywall. It also dramatically extends the reach of journals beyond researchers in Western countries with well-funded libraries, to researchers working in low- and middle-income countries, and to people who are not researchers at all. These new readers may include health professionals seeking an answer to a clinical question, or they might want to produce a Critically Appraised Topic (CAT) to add to an institutional CATBank.

**Some advantages of open access publishing for various stakeholders.**

Individual patients and members of the public

- Easy to locate and access primary and secondary research results to inform their own health-related decisions or advise friends and family.

Patient groups

- Empowers patient and public involvement groups to engage in reshaping health care or clinical service delivery and get involved in formulating research questions (eg, James Lind Alliance) and how researchers address these questions (eg, INVOLVE).

Health professionals

- Allows health professionals to rapidly access primary or secondary research to answer clinical questions at the point of care and thus deliver more evidence-based care.

Guideline development groups and other evidence-based policy developers

- Reduces the costs and lowers the barriers to producing evidence-based practice guidelines and incorporating evidence into other policies (eg, for health promotion).

Health systems

- Lower cost of incorporating evidence-based thinking into the structure and function of a health system.

Researchers carrying out research

- Allows more frequent searching from the researchers’ desktop of a broader range of literature, thus enhancing multi-disciplinary research and helping researchers stay up to date.

Researchers writing articles

- Gives greater reassurance that their research will be read.

Research funders

- Enhances the uptake of results of the research they fund, reassuring patients and the public that their donations or taxes lead to published results with impact.

Journal publishers

- Widens the reach of journals to low- and middle-income countries and nonresearchers (eg, health professionals, patients, and the public). Promotes faster accumulation of data about article readership, enabling rapid-cycle learning (a Learning Publishing System) and enhanced impact.

However, perhaps the most important new reader category that open access supports is patients and the public, allowing them to access research results to guide their self-management decisions. In my view, open access to research results is probably the most critical factor that has led to the global growth of patient involvement networks that influence clinical science. These include groups such as the National Health Service England’s Patient and Public Voice group for clinical service delivery and the National Institute for Health Research’s INVOLVE group for clinical research.

### Potential Disadvantages of the Open Access Model

However, the open-access model can also lead to several new pitfalls for authors and readers, as well as for publishers (see Textbox 2).

One issue here is fraudulent (formerly labeled as “predatory” [12]) publishers and their journals, which can be defined as “spurious scientific outlets that charge fees for editorial and publishing services that they do not provide” [13]. These services include peer review, author retention of copyright, editorial input, copy editing, and a commitment to making articles available online for the long term [13]. Such journals send spam emails to solicit articles for plausible-sounding journals, which can mislead naive authors into submitting their work to a journal that provides few, if any, of the expected services. In turn, this leads to articles with a low probability of citation or clinical impact, thus contributing to the problem of research waste [14]. Worse, because of the lack of effective peer review, some articles in these journals include results that are incorrect or biased, leading to clinical actions that are at best a waste of time, and at worst are potentially harmful [15]. Evidence for the poor quality of peer review comes from a 2013 study in which a journalist sent a seriously flawed, concocted article to 304 open access journals [16]. A total of 157 (52%) journals accepted the article, with only 36 (12%) providing peer reviews that recognized the article’s flaws, though 16 of those journals would still accept the article [16].

Another characteristic of these journals is their willingness to appoint anyone to their editorial board, sometimes in return for cash. Sorokowski et al submitted a fictitious resume for an unqualified scientist applying for membership of 360 journal editorial boards and were surprised to be accepted by 33% [17]. Shamseer [18] developed an empirically based list of differences between fraudulent and legitimate open access journals, based on a study of over 90 of each type. The fraudulent journals showed the following characteristics:

- Spelling errors on the journal home page (66% fraudulent versus 6% legitimate)
- Distorted or unauthorized images (63% versus 5%)
- Promoting a bogus impact factor, the Index Copernicus (33% versus 3%)
- Unverified editor or editorial board affiliation (73% versus 2%)
- Lower article processing fee (median fee US $100 versus US $1865)

**Some potential disadvantages of the open access publishing model.**

Readers

- Tendency to ignore closed-access articles, which mainly affects older material pre-2000. This may lead to higher rates of research duplication, or to failure to incorporate tested classical theories into the design of digital interventions [8].

Authors

- Fraudulent or so-called “predatory” journals.
- The ability to publish is limited by cost (mean article processing fee in health informatics is €2200 [US $2441] [9]), meaning that unfunded research by MSc or PhD students and early career researchers may not get published.

Researchers

- Pressure from funders to publish in open access publishers rather than those journals in which they know their research might have a more significant impact.

Public and patients

- It may be unclear to those lacking critical appraisal skills which journals publish high-quality material and which are fraudulent/“predatory” publishers, thus leading to the spread of pseudo-science or fake news (eg, the global anti-vax movement) [10].

Publishers

- Article processing fees may act as a barrier to authors from low- and middle-income countries or those who are carrying out unfunded research, leading to a Western bias in journal content.
- Added complexity of payment processing.
- Authors now expect faster response times and better service quality from the journal team as they are paying for the service.
- Some scientific society journals may experience insufficient submissions due to authors declining to pay the article processing fee, and thus declining readership [11].
- A stronger emphasis on journal position in article metric, and impact factor league tables may overrule other publishing values.

As a result, authors need to be vigilant and check the rigor of a journal’s refereeing processes and the quality of published articles before they submit, especially if they are responding to one of the numerous emails soliciting articles. They can also check if their intended journal appears on one of the many white lists of journals that are likely to be genuine, such as the Web of Science Journal citation reports, MEDLINE, or the Directory of Open Access Journals. Some professional bodies also publish whitelists of journals relevant to their areas of interest including, for example:

- The Association of Vision Science Librarians [19]
- The International Committee of Medical Journal Editors’ list of journals that claim to follow its Recommendations for the Conduct, Reporting, Editing, and Publication of Scholarly Work in Medical Journals [20]

Research funders should only support publication in journals that commit to high-quality science, and the National Library of Medicine and other bibliographic databases should continue to reject indexing requests from those journals they judge to be fraudulent. However, bibliographic databases and research funders should not presume that all small independent publishers are fraudulent, which has been a barrier to the acceptance of high-quality journals such as JMIR in the past. Senior researchers should reject offers of gift editorship or board membership, however attractive, from fraudulent journals, as that will give the journal spurious authenticity. However, this may not be the case if they genuinely believe that their influence will lead to better quality publications.

However, researchers face a dilemma when approached by an unknown open access journal to referee an article. On the one hand, they can add authenticity by adding their name to the list of reviewers. On the other, by rejecting a poor-quality article, they can improve the quality of the science that the journal publishes and help its editorial staff to better distinguish good from bad science in the future. However, since one of the hallmarks of fraudulent journals is that they tend to carry out refereeing internally if at all, this dilemma will probably not often occur.

## Some Recent Innovations in Open Access Publishing

There are a several important innovations in open access publishing, one of which, Plan S, has significant potential to disrupt scientific publishing fundamentally [21]. Plan S started as a European initiative to overcome some open access publishing challenges, especially for authors seeking funds for article processing fees (APCs), but it is now gathering traction further afield. Plan S proposes that, by 2021, every research funder member of the organization Coalition S, who is promoting this plan, will require all the research they support to be published in one of a list of supported open access journals. Also, the Consortium will directly fund these journals to publish such articles, subject to specific requirements [21]. While this has many advantages, especially for those authors seeking funds to pay APCs, it will only apply to research funded by members of Coalition S. In the United States this currently only includes the Gates Foundation, while in the United Kingdom, this currently includes UK Research and Innovation (the former Research Councils) and the Wellcome Trust. This will leave most US and UK researchers funded by many other bodies, like smaller medical charities, as well as through their own account and student research. However, in January 2019, the United Kingdom’s National Institute for Health Research, the largest clinical research funder in Europe (with a budget of over €1 billion [approximately US $1.3 billion]), pledged support for Plan S and announced that its current open access policy would be reviewed [22].

While many academic organizations have broadly welcomed these principles, some key concerns about Plan S have been raised by publishers, both large and small. This is because Plan S may mean that some publishers (eg, smaller independent publishers such as JMIR or specialist societies such as European Federation for Medical Informatics, International Medical Interpreters Association, or the American Medical Informatics Association) will be sidelined, as major research funders will not recognize their unique contributions towards supporting emerging disciplines, such as health informatics. However, larger publishers are also concerned, with Plan S being described as, “ballistic” by one commentator and Elsevier’s stock price falling by 13% in autumn 2018 after Plan S was mooted [23]. Even the American Association for the Advancement of Science stated that Plan S “will not support high-quality peer-review, research publication and dissemination,” that it “would disrupt scholarly communications, be a disservice to researchers, and impinge academic freedom,” and that it, “would also be unsustainable for the *Science* family of journals” [24,25]. The Open Access Scholarly Publishers Association stated that Plan S puts smaller and emerging fully open-access publishers at a competitive disadvantage and potentially harms their prospects. Thus, they have been lobbying on behalf of the long tail of small open-access publishers whose needs have been ignored by Coalition S [23]. There is even bigger concern over variants of Plan S, such as Projekt DEAL in Germany [26], in which German funders and libraries are making deals with large publishers (and only large publishers) to force them to transition to open access, eradicating in the process smaller publishers and society journals. If, for example, JMIR is not included in this deal, that would make it harder for German health informatics researchers to submit their work to the highest impact journal in the field. Table 1 lists some potential advantages and disadvantages of Plan S, for the same stakeholder groups as before.

**Table 1:** Some advantages and disadvantages of Plan S for various stakeholders.

Stakeholder	Advantages	Disadvantages
Individual patients and members of the public	Easy to locate and access results to inform their own health-related decisions or advise friends and family.	Will only apply to results of research funded by members of Coalition S.
Patient groups	Empowers PPI^a^ groups to engage in reshaping health care or clinical service delivery and get involved in formulating research questions and in how researchers address these questions.	Will only apply to research funded by members of Coalition S.
Health professionals	Allows health professionals to rapidly access primary or secondary research to answer clinical questions at the point of care and thus deliver more evidence-based care.	Will only apply to results of research funded by members of Coalition S.
Guideline development groups and other evidence-based policy developers	Reduces the costs and lowers the barriers to producing evidence-based practice guidelines and incorporating evidence into other policies (eg, for health promotion).	Will only apply to results of research funded by members of Coalition S.
Health systems	Lower cost of incorporating evidence-based thinking into the structure and function of the health system.	Will only apply to results of research funded by members of Coalition S.
Researchers carrying out research	Allows more frequent search from the researchers’ desktop of a broader range of literature, thus enhancing multi-disciplinary research and helping researchers stay up to date.	Will only apply to research funded by members of Coalition S, excluding work funded by smaller organizations and unfunded or student research. May even threaten the existence of some academic disciplines, such as health informatics.
Researchers writing articles	Gives greater reassurance that their research will be read.	Will only apply to researchers funded by members of Coalition S.
Research funders	Enhances the uptake of results of the research they fund, reassuring patients and the public that their donations or taxes lead to published results with impact.	Will only apply to research funded by members of Coalition S.
Journal publishers	Widens the readership of some journals to low- and middle-income countries and nonresearchers. Provides a reliable income stream from Coalition S to journals.	Funding from Consortium S will be subject to meeting several requirements, some of which may be challenging. Likely to favor large, established publishers; could add significant barriers to market entry or growth for small or new publishers, ultimately eradicating smaller publishers and society journals.

^a^PPI: patient and public involvement.

A second innovation for open access is publishing the details of an algorithm (eg, the R syntax or pseudocode) alongside the article which describes its development and validation [27]. This is part of the global movement to “Mobilise Computable Biomedical Knowledge” (MBCK) [28] and was developed analogously with the practice in bioinformatics of publishing gene and protein sequences alongside the article describing their discovery. A third innovation, designed to incentivize reviewers and recognize the effort that peer review takes, is to reduce the APC for peer reviewers through "Karma credits", which was pioneered by JMIR [29]. Ultimately, APCs may disappear as other organizations, such as universities or research funders, pick up the bill for scientific publishing, as proposed by Plan S.

A final innovation with even wider potential consequences is JMIRx [30]. This novel approach inverts scientific publishing so that instead of the author seeking a journal and submitting their article, journal editors scan preprint servers and other sources of research content and contact authors of interesting material requesting a journal-ready article [30].

## Conclusions

There is no doubt that, since JMIR pioneered this new publishing model 20 years ago, open access has led to many benefits for different stakeholders and even opened up new areas of activity, such as patient and public involvement in research and self-management. Of course, open access has led to side effects and unintended consequences, such as the growth of fraudulent journals, but it is now clear that the significance of this challenge has been overstated. More importantly, several recent innovations described above, led by respected brands, such as JMIR, with its impressive record of accomplishment and exciting plans for the future, build on the open-access model and demonstrate its continuing importance and value.

Thinking about how a new open source publisher and editor might ensure that the advantages of open access outweigh its disadvantages for their journal, some suggested principles include: (1) agree with all staff and funders that the core purpose of the journal is help identify, promote, and disseminate high-quality research and its application to improve society and the environment; (2) develop a business model and partnerships that build brand reputation and encourage scientific rigor, originality, and integrity in pursuit of the core purpose, which needs to take higher priority than commercial profit or other short-term considerations; (3) make details of the article review and publishing process, including peer review criteria and scores, open to both authors and readers, and preferably to automated search agents; (4) lower cost barriers to authors where possible, especially to students, early career researchers, and others carrying out unfunded research, using a range of strategies to reduce or eliminate APCs; (5) lower the barriers to readers, especially members of the public, clinicians, and those in low- and middle-income countries, to help them easily locate and access as much of the content as possible; and (6) strive to ensure that all content is well indexed by the major bibliographic services as soon as possible, and that content is retained online long term in third party archives, such as PMC.

Finally, we should encourage all scientific publishers, whether subscription only, open access or hybrid, to develop, test, and share publishing innovations to support the principles outlined above, in the way that JMIR has so clearly demonstrated in its first two decades.

## Abbreviations

APC: article processing charge
CAT: critically appraised topic
JMIR: Journal of Medical Internet Research
MBCK: Mobilise Computable Biomedical Knowledge
